# Extraperitoneal Laparoscopic Radical Prostatectomy and Simultaneously Inguinal Hernia Repair with 3 Trocars

**DOI:** 10.1590/S1677-5538.IBJU.2019.0019

**Published:** 2020-01-10

**Authors:** Yigit Akin, Rodrigo Leon Mar, Sakip Erturhan, Osman Kose, Sacit Nuri Gorgel

**Affiliations:** 1 Department of Urology Izmir Katip Celebi University School of Medicine Izmir Turkey Department of Urology, Izmir Katip Celebi University School of Medicine, Izmir, Turkey;; 2 Department of Urology Centro Medico Nacional Siglo XXI’ Mexico City Mexico Department of Urology, Centro Medico Nacional Siglo XXI’ da médico de base Urología, Mexico City, Mexico;; 3 Department of Urology Gaziantep University School of Medicine Gaziantep Turkey Department of Urology, Gaziantep University School of Medicine, Gaziantep, Turkey

## Abstract

**Objectives:**

Extraperitoneal laparoscopic radical prostatectomy (eLRP) is one of minimally invasive surgical treatment modalities of prostate cancer (PCa). We here evaluated our clinical data on eLRP with 3 trocars.

**Materials and Methods:**

All consecutive patients undergoing eLRP in similar surgical procedures by two different surgeons (Y.A. & R.L.M.) between 2017 and 2018 at two different centres were included. Various clinical data including patients’ demographics, intraoperative and postoperative data, complications, and follow-up were recorded and analysed. We are presenting a video of one such case of eLRP with simultaneously right inguinal hernia repair with using 3 trocars. Surgeons in different clinics performed similar modified surgical technique (Heilbronn technique) (1) for eLRP with 3 trocars.

**Results:**

There were 28 cases in total (10 cases in Clinic 1 and 18 cases in Clinic 2). Mean follow-up was 8±0.4 months. Mean age was 69±3.2 years old. Mean prostate specific antigen was 8.8±1.2ng/dl. Mean operating time was 128±20.2 min. Mean estimated blood loss was 195±87.5 ml. Mean hospitalization day was 2.8±1 days. Mean catheter removal day was 8.8±3.6 days. Surgical margin was negative for all cases with 3 complications (Clavien 2) without major complications.

**Conclusions:**

The first advantage of this technique is using just one assistant holding laparoscopic optic. Second one is to have similar results with traditional eLRP with fewer trocars. Indirectly, more cosmesis is third one, as our approach seems more minimally invasive than traditional eLRP. eLRP with simultaneously hernia repair is feasible, safe, and effective with using just 3 trocars in selected cases.

Available at: http://www.intbrazjurol.com.br/video-section/20190019_Akin_et_al
